# Pre-Heating Effect on Monomer Elution and Degree of Conversion of Contemporary and Thermoviscous Bulk-Fill Resin-Based Dental Composites

**DOI:** 10.3390/polym13203599

**Published:** 2021-10-19

**Authors:** Dóra Kincses, Katalin Böddi, Zsuzsanna Őri, Bálint Viktor Lovász, Sára Jeges, József Szalma, Sándor Kunsági-Máté, Edina Lempel

**Affiliations:** 1 Department of Restorative Dentistry and Periodontology, University of Pécs Medical School, Dischka Gy. Street 5, 7621 Pécs, Hungary; kincses.dora@pte.hu; 2 Department of Biochemistry and Medical Chemistry, University of Pécs Medical School, Szigeti Street 12, 7624 Pécs, Hungary; katalin.boddi@aok.pte.hu; 3 Department of Physical Chemistry and Materials Science, University of Pécs, Ifjúság Street 6, 7624 Pécs, Hungary; orizsuzsa@gmail.com; 4 János Szentágothai Research Center, Ifjúság Street 20, 7624 Pécs, Hungary; sandor.kunsagi-mate@aok.pte.hu; 5 Department of Oral and Maxillofacial Surgery, University of Pécs Medical School, Dischka Gy. Street 5, 7621 Pécs, Hungary; balint10@hotmail.co.uk (B.V.L.); szalma.jozsef@pte.hu (J.S.); 6 Faculty of Sciences, University of Pécs, Ifjúság Street 12, 7624 Pécs, Hungary; sara.jeges@etk.pte.hu; 7 Institute of Organic and Medicinal Chemistry, Faculty of Pharmacy, University of Pécs Medical School, Szigeti Street 12, 7624 Pécs, Hungary

**Keywords:** bulk-fill, pre-heating, degree of conversion, monomer elution

## Abstract

Detection of unreacted monomers from pre-heated resin-based dental composites (RBC) is not a well-investigated topic so far. The objectives were to determine the temperature changes during the application and polymerization, the degree of conversion (DC) and unreacted monomer elution of room temperature (RT), and pre-heated thermoviscous [VisCalor Bulk(VCB)] and high-viscosity full-body contemporary [Filtek One Bulk(FOB)] bulk-fill RBCs. The RBCs’ temperatures during the sample preparation were recorded with a K-type thermocouple. The DC at the top and bottom was measured with micro-Raman spectroscopy and the amounts of eluted BisGMA, UDMA, DDMA, and TEGDMA were assessed with High-Performance Liquid Chromatography. The temperatures of the pre-heated RBCs decreased rapidly during the manipulation phase. The temperature rise during photopolymerization reflects the bottom DCs. The differences in DC% between the top and the bottom were significant. RT VCB had a lower DC% compared to FOB. Pre-heating did not influence the DC, except on the bottom surface of FOB where a significant decrease was measured. Pre-heating significantly decreased the elution of BisGMA, UDMA, DDMA in the case of FOB, meanwhile, it had no effect on monomer release from VCB, except TEGDMA, which elution was decreased. In comparison, RBC composition had a stronger influence on DC and monomer elution, than pre-cure temperature.

## 1. Introduction

During the restorative procedure, it is favorable to use an easy-to-handle, non-technique sensitive, durable and esthetic restorative material with quick and efficient polymerization. In the case of resin-based composite (RBC) restorations, expediting polymerization, increasing of maximum layer thickness, and the degree of monomer conversion can be considered as the main objectives [1]. To achieve a durable, successful composite restoration, the most important factors, among others, are mechanical properties, handling characteristics, polymerization stress, marginal adaptation, and degree of polymerization [2].

According to the literature data, there is a clear correlation between the degree of conversion (DC) and the physicochemical characteristics of RBCs [3,4]. Meanwhile, DC is influenced by several factors, such as light exposure conditions, composition, shade, opacity, and thickness of the RBC, pre-cure temperature also plays an important role in the polymerization process [5,6]. As the success of RBC restorations depends on their polymerization and DC, the influence of temperature has become one of the central issues of several studies [7,8].

The polymerization of RBCs is an exothermic reaction and the released heat is mainly produced in the propagation phase [9]. The process of monomer conversion and the properties of the set polymer are influenced by the polymerization temperature [10]. An elevation in temperature promotes molecular mobility and increases the collision frequency of reactive radicals, resulting in higher conversion and delayed auto deceleration [6,9]. Not only the exothermic reaction but also the light energy absorbed contributes to the system temperature during the polymerization of light-cured RBCs [11]. While some authors attribute a greater significance to the heat emitted by the light-curing unit in the temperature rise, others regard the heat generated in the exothermic reaction as more important [12,13].

As reported by several investigations, pre-heating may have a beneficial impact on marginal adaptation, gap formation, and microleakage by reducing the viscosity of RBCs [7,14,15]. Improved handling properties, such as flowability can facilitate the application of the filling material, consequently making the procedure less time-consuming. Reduced viscosity also improves marginal seal thereby contributing further to the overall clinical success [16,17]. There may be a lack of efficiency associated with the use of conventional heating devices as some authors have shown a rapid decrease in RBC temperature after removal of the device, as well as during dispensing and handling [2,18]. Moreover, during the cooling phase, the system bears a loss of energy, so vitrification takes place earlier and causes decreased DC [19].

Several types of RBC dental materials have been developed over the years, including bulk-fill RBCs, which can be placed in larger increments to reduce operating time and technique sensitivity [20]. The primary advantage of bulk-fill RBCs over conventional ones is the increased depth of cure [21,22]. According to a literature review by Van Ende et al., the maximum layer thickness which still ensures adequate material characteristics as recommended by the manufacturers, is 4 mm or in some cases even 5 mm. Although most studies have confirmed the improved depth of cure for bulk-fill RBCs, some controversial data can still be found [23,24]. Besides the DC, polymerization shrinkage stress is another important issue addressing the clinical failures. Ausiello et al. demonstrated, that bulk-filling, especially in deep cavities induces higher shrinkage stress along the cavity walls compared to a multilayer technique (i.e., no shrinking glass-ionomer basing and shrinking bulk-fill RBC cover) [25].

Recently, a thermoviscous bulk-fill RBC (VisCalor Bulk) and a new heating device (VisCalor Dispenser) were introduced to combine the advantages of bulk-fills and pre-heating. This delivery system can warm up the filling material in seconds using near-infrared technology and allows immediate application without removal of the capsule from the heating device thereby maintaining its increased temperature [26]. Although VisCalor Bulk is a relatively new RBC, it has already been the subject of several investigations [27,28,29,30,31]. Yang et al. examined the effects of temperature on stickiness and packability, and the effect of pre-heating time on pre-cure properties. The study found pre-heating to lead to a reduced extrusion force and increased flowability without premature polymerization, while stickiness and packability remained within a clinically acceptable range [27].

The effect of pre-heating and exposure duration on other properties of VisCalor Bulk has been investigated in another study undertaken by Yang et al. This article reported a longer exposure duration not to have an effect on the degree of conversion, maximum rate of polymerization and polymerization shrinkage, however, it did lead to an increase top surface microhardness. The application of 3 min pre-heating and 20 s irradiation provided adequate hardness without unfavorable changes in polymerization shrinkage strain and polymerization kinetics [28]. The study of Marcondes et al. examined viscosity and thermal kinetics of pre-heated RBCs including VisCalor Bulk as well as the effect of ultrasound energy on film thickness. VisCalor Bulk showed the greatest extent of viscosity reduction at 69 °C, while film thickness could not be reduced below 50 μm without the use of ultrasound. This study also claimed that to take full advantage of the pre-heated RBCs, the ideal working time is merely 10–15 s [29]. Demirel et al. investigated the effect of different insertion techniques on the internal void formation and found VisCalor Bulk to show the lowest void percentage with the utilization of the pre-heating technique [30]. Colombo et al. evaluated microhardness and depth of cure of four bulk-fill RBCs. According to the measured ratio of top to bottom hardness, all tested materials—including VisCalor Bulk—showed an adequate degree of polymerization. In addition, in the case of VisCalor Bulk, acid storage led to one of the highest mean percentage losses in micro-hardness of the external side [31].

Besides the physicomechanical properties, the chemical characteristics are also important determinants of the clinical performance and biocompatibility of an RBC [32]. Although there is a strong inverse correlation between DC and monomer elution, the number of released monomers may be influenced further by other factors such as the quality of the monomer system, filler type, content, porosity as well as employed solvent [24,33]. Elution from bulk-fill RBCs was found to be comparable to that of conventional materials despite their increased increment thickness as monomer release is more dependent on the hydrophobicity of the base monomers and the final network characteristics of the resin-matrix [34]. Detection of unreacted monomers from pre-heated RBCs is not a well-investigated topic so far. To the best of our knowledge, the current literature has no information regarding the amount of eluted monomers from thermoviscous VisCalor Bulk.

Therefore, the purpose of the present study was to investigate the temperature changes during the application and polymerization of a new thermoviscous (VisCalor Bulk) and a high-viscosity full-body bulk-fill RBC (Filtek One Bulk-fill) in relation to different pre-cure temperatures. Further aims were to evaluate the effect of pre-heating on the degree of conversion (DC) and the number of released monomers using micro-Raman spectroscopy and Reversed-phase High-Performance Liquid Chromatography (RP-HPLC). The Null Hypotheses were: (1) Pre-heating had no effect on RBCs’ post-cure DC%, and (2) pre-cure temperature did not affect the amount of released unreacted monomers.

## 2. Materials and Methods

### 2.1. The Bulk-Fill Resin-Based Composites and Sample Preparation

During this in vitro study two brands of high-viscosity bulk-fill RBCs (Viscalor Bulk and Filtek One Bulk-fill)—A2 shade for both—were investigated in 4 mm layer thickness. The specifications of the materials and their acronym codes are presented in Table 1.

**Scheme 1:**
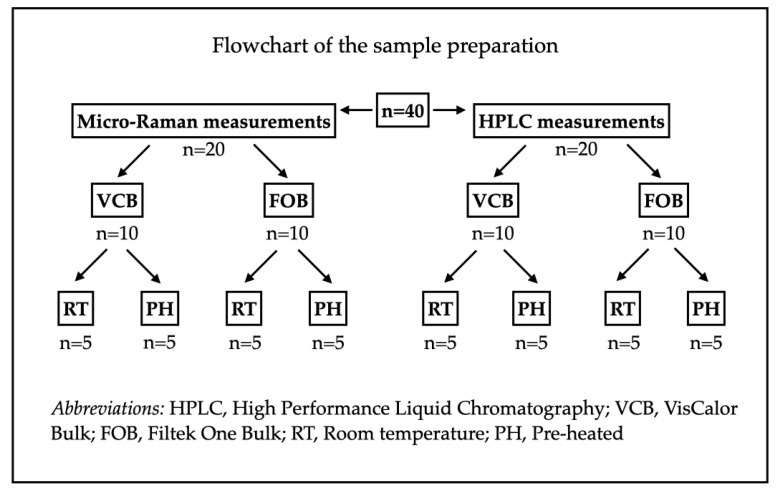
Flowchart of the sample preparation.

According to the method of sample preparation, there were two experimental groups for each of the two investigated materials. The pre-cure temperature of the RBC samples in the first group was 25 °C (room temperature—RT) (FOB_RT and VCB_RT), while RBCs in the second group were preheated before the sample preparation. In the case of VCB, pre-heating was performed by VisCalor Dispenser (VOCO, Cuxhaven, Germany) using T1 setting (VCB_65) (30 s pre-which warmed the device and RBC together to 65 °C). Pre-warming of FOB was undertaken by Ena Heat Composite Heating Conditioner (Micerium, Avegno, Italy) using T2 setting (FOB_55) (55 min pre-warming of the device to 55 °C and 15 min pre-warming of the RBC). Five specimens were prepared in each group, from each material for both the micro-Raman spectroscopy measurements as well as for the monomer elution measurements (Scheme 1).

The samples were prepared in a cylindrical polytetrafluoroethylene (PTFE) mold with an internal diameter of 5 mm and a height of 4 mm, placed on a thermostatically controlled (30 ± 1 °C) glass slide to represent the isolated tooth. A polyester Mylar strip was positioned between the mold and the glass slide. A capsule dispenser gun was used to apply VCB_RT, FOB_RT, and FOB_55 materials into the mold. In the case of VCB_65, a VisCalor dispenser was used for both warming and application. The condensation of the RBCs was performed with a room temperature hand instrument. Before irradiation, the RBC sample was covered with a transparent polyester strip (Mylar, Dentamerica Inc., San Jose Ave, CA, USA) to avoid contact with oxygen. All specimens were irradiated with a Light Emitting Diode (LED) curing unit (LED.D, Woodpecker, Guilin, China; average light output given by the manufacturer 850–1000 mW/cm^2^; ʎ = 420–480 nm; 8 mm exit diameter fiberglass light guide) in standard mode for 20 s, powered by a line cord at room temperature of 25 °C ± 1 °C, controlled by an air conditioner. The irradiance of the LED unit was monitored before and after polymerization with a radiometer (CheckMARC, Bluelight Analytics, Halifax, NS, Canada). The tip of the fiberglass light guide was in direct contact, centrally positioned, and parallel to the mold. All the samples were prepared by one operator. 

### 2.2. Temperature Measurement

**Figure 1:**
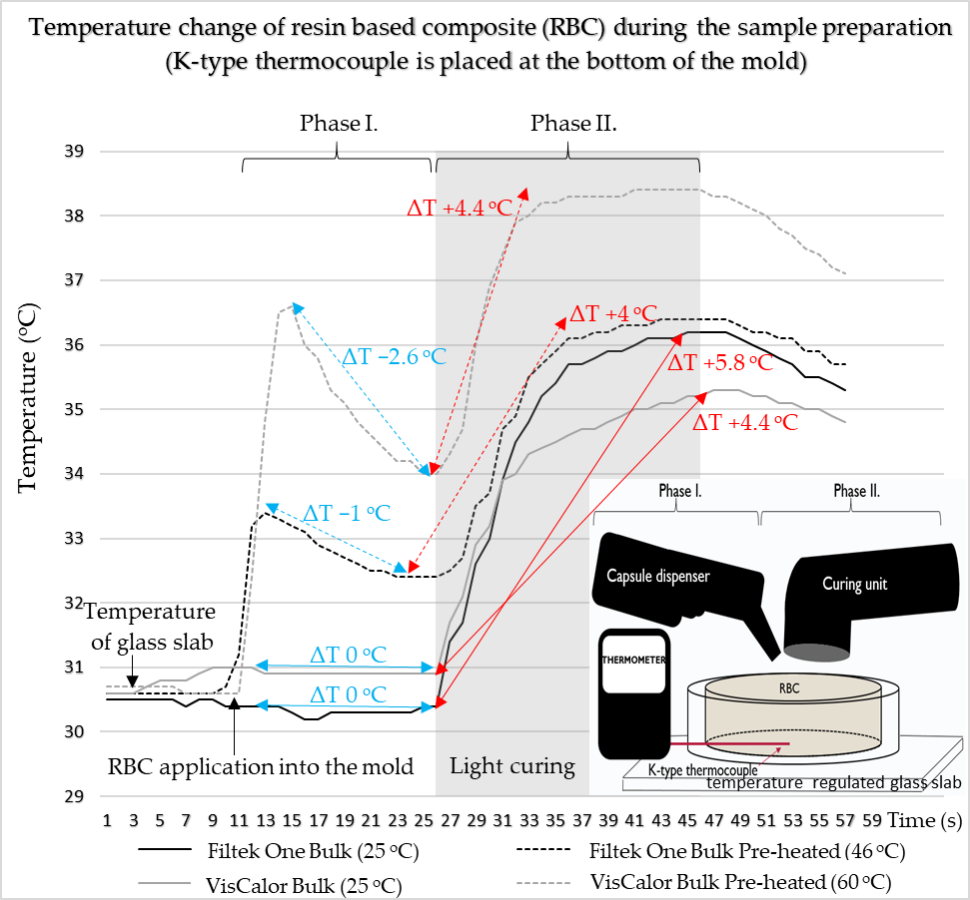
Temperature change of resin-based composite during the sample preparation.

Temperature measurements during the application and the polymerization of RBCs’ were recorded with a registration device (El-EnviroPad-TC, Lascar Electronics Ltd., Salisbury, UK) attached to 0.5 mm diameter Cu/CuNi thermocouple probes (K-type, TC Direct, Budapest, Hungary)—positioned at the bottom of the temperature regulated mold—with a frequency of one measurement per second and resolution of 0.1 °C (Figure 1. schematic component). The quantity of heat emitted by the LED curing unit was also determined through the 4 mm empty mold.

### 2.3. Micro-Raman Spectroscopy Measurement

RBC samples made to estimate the temperature change of the composite during sample preparation were then used to measure the DC. Confocal Raman spectrometer (Labram HR 800, HORIBA Jobin Yvon S.A.S., Longjumeau Cedex, France) was used to evaluate the 24 h post-cure DC values of the polymerized RBC samples. Setting parameters for the measurements were the following: 20 mW He-Ne laser with 632.817 nm wavelength, magnification × 100 (Olympus UK Ltd., London, UK), spatial resolution ~15 µm. The spectral resolution of ~2.5 cm^−1^ provided satisfactory results since the two peaks analyzed were ~30 cm^−1^ apart. Spectra were taken at three locations of the RBC samples (center, periphery, and between these two regions) both from the bottom and top surfaces with an integration time of 10 s. Ten acquisitions were averaged for each geometrical point. Spectra of uncured RBCs were taken as reference. Post-processing and analysis of spectra were performed using the dedicated software LabSpec 5.0 (HORIBA Jobin Yvon S.A.S., Longjumeau Cedex, France) [13]. The ratio of double-bond content of monomer to polymer in the RBC was calculated according to the following equation:

DC%=(1−(RcuredRuncured))×100

where *R* is the ratio of peak intensities at 1639 cm^−1^ and 1609 cm^−1^ associated with the aliphatic and aromatic (unconjugated and conjugated) C=C bonds in cured and uncured RBCs, respectively.

### 2.4. Reversed-Phase High-Performance Liquid Chromatography Measurement 

Immediately after the irradiation, five samples of each material were immersed into 1.0 mL of the 75% ethanol/water storage medium in separate glass vials and stored in a 37 °C incubator. As recommended by the ISO 10993-13 description, the ratio between the sample and the storage solution volume was greater than 1:10, thus the specimens were fully immersed in the medium. The storage solutions were collected for analysis after 72 h. The RP-HPLC system (Dionex Ultimate 3000, Thermo Fisher Scientific Inc., Sunnyvale, CA, USA) consists of a Dionex LPG 3400 SD gradient pump, Rheodyne injector (Rheodyne, CA, USA), and a Dionex DAD 3000 RS UV–VIS detector (Dionex GmbH, Germering, Germany). Data acquisition was completed using Chromeleon software (version: 7.2.10). The separations were performed on a LiChrospher^®^ 100 RP-18e (particle size: 5 μm, pore size: 100 Å) (Merck KGaA, Darmstadt, Germany) column (250 mm × 4.00 mm) with gradient elution. The composition of Eluent “A” was 100% bidistilled water, whereas Mobile Phase “B” was 100% *v/v* acetonitrile (ACN) (VWR International, Radnor, PA, USA). During the 30-min chromatographic separation, the “B” eluent content increased from 30–95%. The flow rate was 1.2 mL × min^−1^. For the regeneration of the stationary phase, the content of Mobile Phase B was decreased from 95% to 30% in 1 min, and after 31–46 min, the system was washed with 30% “A”. The detection of the eluted monomers was carried out at the following wavelengths: 205, 215, 227, and 254 nm. 205 nm were found to be optimal; therefore, the evaluation relied on the data collected at this wavelength [24]. The separations were undertaken at room temperature. The amounts of the eluted monomers (Bisphenol A-glycidyl methacrylate, BisGMA; Trietylene-glycol-dimethacrylate, TEGDMA; urethane-dimethacrylate, UDMA; 1,12-dodecanediol-dimethacrylate, DDMA) were calculated using the calibration curve with the areas under the curve of peaks produced by the monomers, respectively. The monomer release was counted to 1 mg RBC. The TEGDMA, UDMA, BisGMA, and DDMA standard solutions had retention times of 12.2, 17.2, 19.1, and 27.2 min, respectively, whereas the peaks were well separated from each other.

### 2.5. Statistical Analysis

Pilot study results and sample size formula were used to estimate sample size. 

Sample size formula: $n=\frac{{\left(z_{1-\frac{\alpha }{2}}+z_{1-\beta }\right)}^{2}{\left(s_{1}+s_{2}\right)}^{2}}{{\left(M_{1}+M_{2}\right)}^{2}}$ = 3.

(*z* = standard score; α = probability of Type I error = 0.05; *z*_1−α/2_ = 1.96; *β* = probability of Type II error = 0.20; 1 − *β* = the power of the test = 0.80; *z*_1−*β*_ = 1.28, *M*_1_ = 52, *s*_1_ = 1.4, *M*_2_ = 52, *s*_2_ = 1.4). By adopting an alpha (α) level of 0.05 and a beta (*β*) level of 0.20 (power = 80%), the predicted sample size (*n*) was found to be a total of 3 samples per group. Instead of the calculated 3 samples, *n* = 5 per group sample size was selected.

The statistical analyses were performed with SPSS v. 26.0 (SPSS, Chicago, IL, USA). Levene’s test was employed to test the equality of variance. This was followed by Paired Samples Test to analyze the differences in mean DC% between top and bottom surfaces and Two-tailed Independent Samples T-test to analyze the differences in mean DC% between the investigated materials polymerized at room temperature and with the application of pre-heating. The differences in monomer elution from the RBCs at the investigated temperatures were also compared with the Two-tailed Independent T-test.

Multivariate analysis (General Linear Model) and Partial Eta-Squared statistics were used to test the influence and describe the relative effect size for Material and Temperature as independent factors. *p* values below 0.05 were considered statistically significant.

## 3. Results

The measured maximum radiant exitance of the LED LCU was 1250 ± 15 mW/cm^2^. The delivered radiant exposure was 25 J/cm^2^. The LCU increased the temperature by an average of 7 °C when the thermocouple was irradiated through the empty 4 mm deep mold for 20 s. 

Meanwhile, the Ena Heat Composite Heating Conditioner T2 setting stated preset temperature is 55 °C in 55 s, the real temperature of the FOB was 46 °C in the capsule after the recommended pre-warming period. The VisCalor Dispenser T1 setting provides 65 °C pre-warming in 30 s, however, the actual temperature of VCB was 60 °C after the recommended duration of pre-heating. Figure 1 shows the temperature change of RBC during the sample preparation from the material application into the mold until the end of the polymerization. The temperature of the 46 °C pre-heated FOB decreased to 33.4 °C as it was removed from the warming device and started to be applied into the mold and showed further temperature drop of −1 °C during the condensation. The total temperature decrease from the pre-heating until the start of polymerization was 13.6 °C in approximately 20 s. In the second phase, during polymerization, the exothermic reaction and the heat released from the curing unit elevated the RBC’s temperature by 4 °C. Regarding the thermoviscous VCB, its pre-heated temperature (60 °C) decreased to 36.6 °C during the initial phase of the application and continued to show a further drop of (−2.6 °C) during the condensation phase The total drop of temperature for the pre-heated VisCalor Bulk from the pre-heating until the start of polymerization was 26 °C in approximately 20 s. The temperature rise caused by the light-curing and the exothermic reaction was 4.4 °C. The consistency of the room temperature materials was highly viscous, especially of Viscalor making it difficult to squeeze out of the capsule. Compressing both room temperature RBCs was easy without sticking to the instruments. The pre-heating decreased the viscosity to a flowable consistency which allowed both materials to spread evenly throughout the template.

Considering the DC at the top and bottom surfaces in samples applied in 4 mm thickness, the mean percentages ranged between 54.2–64% and 45.0–51.8%, respectively (Table 2). 

**Figure 2:**
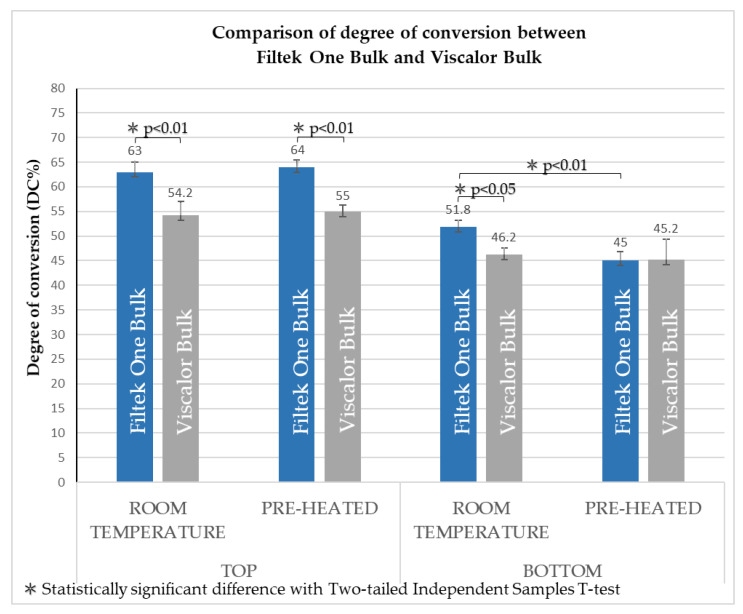
Comparison of the degree of conversion between Filtek One Bulk and Viscalor Bulk.

The DC values at the bottom of the specimens showed a statistically significant decrease for both materials at both temperatures in relation to the DC values measured at the top of the samples. When room temperature specimens were applied, the DC values were very similar to that of preheated samples, except on the bottom surface of FOB which was significantly lower when applied after pre-heating. In a comparison of the two bulk-fill RBCs, VCB showed a statistically significantly lower DC (~10% less) both on the top and bottom when applied at room temperature. Samples applied following pre-heating showed a significantly lower DC% only on the top (Figure 2). The lowest DC values were measured on the bottom surfaces of both investigated RBCs when they were applied with pre-heating. 

Table 3 presents the relative effect size of factor Material, Temperature, and their interactions on the degree of conversion of the top and bottom surfaces of the investigated resin-based composites. 

A 2 (Material) × 2 (Temperature) mixed-model ANOVA revealed that the main effect for Material on DC values measured on top surfaces was significant and the Partial Eta-squared was considered to be large, meanwhile not significant effect for Temperature was obtained with medium effect size. The interaction (Material × Temperature) had no effect on the monomer conversion at the top. 

Regarding the DC values at the bottom surfaces, the results showed a significant effect for both the Material and Temperature factor. The interaction between the two variables (Material × Temperature) also significantly affected the monomer conversion at the bottom surfaces. The main effect for Material was significant at room temperature, meanwhile, the Temperature factor affected significantly only the FOB RBC.

In addition to the monomers specified by the manufacturers, other methacrylates were also detected from both FOB (BisGMA) and VCB (TEGDMA, DDMA) RBCs. 

**Figure 3:**
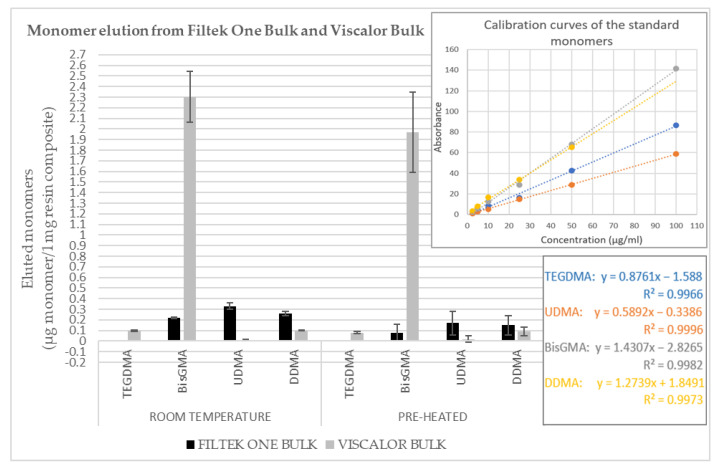
Monomer elution from Filtek One Bulk and Viscalor Bulk.

The differences in the monomer elutions were also significant between FOB and VCB both when applied at room temperature and with pre-heating in the case of all the evaluated monomers, except for DDMA which was released in similar (statistically insignificant) amounts from the pre-heated RBCs (Table 4 and Figure 3).

At room temperature, 30 and 2.5 times as much UDMA and DDMA were released, respectively from FOB, while 10.5 times more BisGMA was eluted from VCB. The latter was the monomer released in the largest amount. With the utilization of pre-heating, 7.5 times as much UDMA was found to elute from FOB, while 25 times more BisGMA was released from VCB. For FOB, preheating significantly reduced the amount of eluted monomers, while for VCB, the temperature did not affect the dissolution (Table 5).

The following order of mean monomer elution was detected from FOB for both room temperature and pre-heated samples from highest to lowest: UDMA < DDMA < BisGMA, meanwhile the amount of leached monomers was roughly half (UDMA, DDMA) or one-third (BisGMA) for pre-heated specimens.

Regarding VCB, both the order, as well as the amount of the released monomers were the same in the case of both the room temperature and the pre-heated samples (BisGMA < TEGDMA < DDMA < UDMA), except for TEGDMA, which showed a significantly lower elution from the pre-heated samples. 

Table 6 presents the relative effect size of factor Material, Temperature, and their interactions on the monomer elution from the investigated resin-based composites.

A 2 (Material) × 2 (Temperature) mixed-model ANOVA showed that the main effect for Material was significant on UDMA, BisGMA, DDMA release with a Partial Eta-squared value which was considered to be large. The Temperature factor also influenced significantly the monomer elution, however, its effect was slightly weaker compared to the Material’s effect. TEGDMA was released only from VCB. The effect of the Temperature factor was calculated to be significant on the elution of this monomer. The interaction between the two factors (Material × Temperature) had a significant effect on UDMA and DDMA elution, while the elution of BisGMA was independent of the Material × Temperature interaction.

## 4. Discussion

In this in vitro study, the DC and the elution of unreacted monomers of a thermoviscous and high-viscosity bulk-fill dental resin composites were assessed using micro-Raman spectroscopy and High-Performance Liquid Chromatography measurements. Additionally, the thermal change of the RBCs was also registered during the sample preparation with a K-type thermocouple to assess the temperature change of the room temperature and pre-heated RBCs during the manipulation and polymerization phases.

The setting reaction of RBCs has a major influence on their mechanical and biological properties [35]. RBC polymerization depends mainly on the chemical structure of the monomers, filler characteristics, the photoinitiator type, and concentration, and the polymerization conditions [36]. The latter includes, among others, the volume and the layer thickness of the applied RBC, spectral characteristics of the curing unit, exposure time, and the pre-cure temperature of the material [4,14,37]. Since these were standardized in this study, except for pre-cure temperature, differences in the DC value and monomer elution of the bulk-fill RBCs can be attributed to the different compositions and temperatures of the materials before polymerization. Thus, the effect size of the material factor and pre-cure temperature factor became assessable on the degree of conversion and monomer elution from the investigated bulk-fill RBCs. 

According to our results, the first null hypothesis, which stated that the pre-heating had no effect on post-cure DC% of VisCalor Bulk and Filtek One Bulk, was partially rejected, since pre-warming of RBCs neither increased nor decreased the DC on the top of both materials and the bottom of VCB_65, however, the bottom DC was significantly decreased in the case of FOB_65. The second null hypothesis was also partially rejected, because the external heating of the investigated RBCs decreased the monomer elution in the case of FOB regarding all the investigated monomers and TEGDMA elution VCB, however, had no influence on the BisGMA, UDMA, and DDMA release from VCB. 

It has been reported that increased pre-cure temperature of RBC may result in a greater extent of monomer to polymer conversion [6,38]. However, investigations, that have shown improvement in the degree of polymerization upon pre-warming generally maintained the RBC temperature constant during the experimentation [6,10]. On the other hand, there are also results that found increases in DC at non-isothermal conditions to be material composition-dependent [19,39]. 

Regarding the real-life clinical scenario, the RBC’s temperature drops rapidly to the physiological level upon removal from the pre-heating device [2,29]. In contrast to the studies that have demonstrated optimized monomer conversion in the case of pre-heated RBCs under isothermal conditions, Yang et al. and Tauböck et al. reported that the pre-heated RBC’s temperature dropped to ~35–36 °C during the handling phase before light-cure [27,39]. Additionally, the pre-warmed RBC can reach a lower internal temperature than the maximum stated preset temperature of the heating device [27,40].

To overcome this problem, a new warming device was developed, namely, VisCalor Dispenser. The capsule dispenser itself can provide homogeneous warming of the highly filled RBCs to 68 °C (only for VOCO products) with near-infrared technology. Thus, the RBC does not need to be removed from the heating device for dispensing into the prepared cavity. During application it is flowable and when it comes in contact with the tooth VCB reaches body temperature within a short time and thus returns to the high-viscosity, sculptable state. 

In the present study, during the specimen preparation, the glass slab, holding the PTFE mold, was pre-set to a temperature of 30 ± 1 °C, representing a rubber dam isolated tooth [41]. In the case of both investigated materials, the temperature decreased during the extrusion from the capsule, irrespectively to the type of the heating device, and a further drop of temperature was observed during the condensation into the mold (Phase I on Figure 1). The measured temperatures for FOB_55 and VCB_65 were on average 32.5 °C and 34 °C, respectively, at the start of polymerization. The direct contact to the 30 °C molds and glass slab and also to the room temperature condensing instrument accelerated the cooling of the RBCs. The equilibration of the ambient and the pre-heated RBC’s temperature resulted in faster cooling of the warmer RBC. During the photopolymerization (Phase II on Figure 1.), both exothermic reaction and the released heat from the curing device increased the RBCs’ temperature. The extent of temperature increase, however, seems to be influenced by the speed of the temperature drop in Phase I. Accelerated drop may hinder the exothermic temperature increase. 

Adequate monomer to polymer conversion is crucial to the material’s long-term clinical success [42]. While DC is the key parameter determining the effectiveness of monomer conversion, unfortunately, it cannot describe the microstructure of the resulted heterogenic polymer network, which has a major effect on the physical and chemical properties of the RBC [43]. To determine the DC, micro-Raman spectroscopy was used in our study. It offers the possibility of quantitative characterization of the polymerization extent in dimethacrylate-based RBCs [44]. Raman-spectra were taken after 24 h, since a significant increase in DC takes place during the 24 h post-irradiation [28]. Although the minimum DC% for clinically acceptable restoration has not yet been precisely defined [45,46], DC values below 55% may be inadequate for occlusal restorative layers [47,48]. Musanje and Darvell recommended that the depth of cure should be defined as the depth at which maximum conversion occurs for a given irradiance and exposure time [49]. The radiant exposure was defined at 16–24 J/cm^2^ to reach an adequate polymerization degree for a 2 mm thick RBC layer [50,51], meanwhile, the minimum radiant exposure required to be delivered to different bulk-fill RBCs moves on a wider scale (14–23–47 J/cm^2^) [52,53]. In our study, the valid, portable radiometer measured a higher value of radiant exitance than the average light output given by the manufacturer. It was demonstrated that most of the LCUs—especially low-budget LCUs, like our LED.D—could have different light output characteristics [54]. Providing by the curing unit, the delivered radiant exposure was 25 J/cm^2^, and the DC% on the top of FOB_RT and FOB_55 were 63% and 64%, respectively, which is a characteristic value for a well polymerized RBC [49]. In comparison to the FOB values, the DC% on the top of the VCB_RT and VCB_65 samples were significantly lower, 54% and 55%, respectively. The lower DC values are presumably due to the material composition. The monomer system has a major effect on the DC, which increases in the following order: BisGMA < BisEMA < UDMA < TEGDMA [55]. BisGMA is considered to be the most viscous monomer due to the strong intramolecular hydrogen bonding, resulting in limited rotational freedom, thus the reactivity and mobility of the monomer may decrease during the polymerization process [56]. This might be one of the explanations for the significantly lower DC of the VCB, which is a BisGMA-based RBC. FOB is an UDMA-based bulk-fill RBC, containing both aliphatic and aromatic UDMA. Sideridou et al. found that UDMA, combining relatively high molecular weight with a high concentration of double bonds and low viscosity, was shown to reach higher final DC% values than BisGMA [55]. Although the viscosity of UDMA is much lower than that of BisGMA, when it is mixed with the high molecular weight BisGMA or BisEMA, it can significantly restrict the mobility of UDMA monomers and decrease their reactivity and conversion value [57,58]. In addition to the monomer system, the filler-matrix ratio is also decisive. VCB filler loading is higher (83 wt%) compared to the filler content of FOB (76.5 wt%), which may restrict the light penetration and the mobility of monomers and radicals. The DC of VCB was investigated by Yang et al., and their results showed similar values (~58 DC%) in a 2 mm thick sample, exposed either with 20 s (24 J/cm^2^) or 40 s (48 J/cm^2^) [28]. Similar to the above study, pre-heating did not influence the DC% values of our investigated materials at the top of the samples, assuming that the RBCs on the top reached their maximum conversion degree already at room temperature. Although Daronch et al. found that the increased pre-cure temperature significantly improved the DC compared to the room temperature, they also concluded, that at longer exposure (20 s, 40 s) top-surface composite conversion was equivalent and similar throughout the tested temperature range (22–60 °C) [10].

Contrary to the values measured at the top of the room temperature samples the DC% at the bottom of the 4 mm thick bulk-fill materials were lower by ~10% (FOB_RT, 51.8%; VCB_RT, 46.2%). While the depth of cure is improved in bulk-fills due to increased translucency, modified matrix composition, photoinitiator kinetics, and filler characteristics, not all of the commercial bulk-fill RBC are able to sustain a homogeneous conversion at a depth of 4 mm [24,59,60,61]. The present research examined two, so-called full-body bulk-fill RBCs that are often referred to as paste-like bulk-fills. These materials generally have a higher filler load which makes them highly viscous and therefore sculptable. The higher filler content renders the surface more wear-resistant without requiring any coverage. Decreased DOC from the surface to the bottom may be a result of the increased filler ratio which may hinder light penetration due to the nano-sized particles despite the increase in translucency. On the other hand, high molecular weight monomers, such as BisGMA (in VCB) and aromatic UDMA (in FOB), also help to increase the viscosity, however, decreasing the reactive groups in the resin may negatively influence the DC [56,62].

Pre-heating makes highly filled, sculptable RBCs more flowable, adaptable, and easier to manipulate, without compromising the superior mechanical properties. Decreased viscosity has been shown to enhance marginal adaptation and reduce microleakage due to improved wetting of cavity walls [14,15]. Although, the increased pre-cure temperature has benefits through decreased system viscosity, enhanced radical mobility, and collision frequency of unreacted active groups resulting in additional polymerization and higher conversion [63], the diversity of study outcomes may result from different RBC composition and experimental set-ups. Isothermal conditions mostly favor the positive effect of pre-heating on monomer conversion resulting in more highly cross-linked polymer networking and improved mechanical and physical properties [10]. However, improved monomer to polymer conversion has a strong relation to polymerization shrinkage which may increase the shrinkage stress of the bonded restoration [47,63,64]. Despite the higher shrinkage which might be present, it may not be clinically significant, as it can be offset by the improved marginal adaptation [63].

Clinically relevant, non-isothermal circumstances enhance the strong effect of RBC composition on the results. Several studies found the effect of pre-heating to vary on DC (decrease, no change, increase) depending on the composition of the investigated RBC [14,19,39]. 

Confirming the above findings, our results also showed a dissimilar effect of pre-heating on the monomer conversion of the investigated RBCs. An increase in pre-cure temperature did not influence significantly the DC on the top surfaces neither for FOB_55 nor for VCB_65 and even did not affect the bottom DC of VCB_65 compared to the room temperature RBCs. The bottom DC values showed a significant decrease however in the case of FOB_55. The rapid temperature drop of pre-heated RBC during handling results in excess heat loss which may deprive energy of the system and might prevent a sufficient increase in polymerization reactivity and consequent enhancement in monomer conversion [19]. Considering the findings of the temperature measurements, it is visible, that the temperature increase during polymerization shows a direct correlation with the measured DC values. During polymerization (Phase II. on Figure 1), the temperature of both VCB_RT and VCB_65 increased by 4.4 °C and showed similar DC values at the top (54.2 °C and 55 °C, respectively) and as well on the bottom surfaces (46.2 °C and 45.2 °C, respectively). The mean differences between top and bottom DCs were around 10%. Meanwhile, the temperature within FOB_RT during polymerization rose by 5.8 °C and showed a significantly higher DC both on the top (63 °C) and bottom (51.8 °C), compared to VCB_RT. The mean difference on top vs. bottom DC was found to be 10% also. In contrast, pre-heating of FOB had a negative effect both on the exothermic reaction and on the kinetics of monomer conversion. During light-curing, the temperature rise within FOB_55 was 1.8 °C lower (4 °C) than in the case of FOB_RT, and the bottom DC was 20% less (45%) compared to the top DC value, which kept its higher level (64 °C). Although the drop of temperature during the dispensing and condensation phase (Phase I. on Figure 1) of VCB_65 was rapid, its temperature at the initiation of light-curing was higher, compared to the FOB_55. It may have provided enough energy to the polymerizing system, assuming that even higher exothermic temperature rise and higher DC would have been achieved if the system temperature dropped slower. In contrast, the temperature of FOB_55 at the beginning of light-curing was lower, and the additional drop during the cooling phase may have deprived energy from the system, resulting in a weaker exothermic reaction and lower monomer conversion. Since the reaction behavior of multifunctional monomer systems is very complex and highly dependent on the reaction conditions and composition, other possible explanations for DC decrease may arise. The increased pre-cure temperature may induce thermal polymerization before irradiation. On one hand, thermal polymerization leads to the consumption of functional groups, and on the other hand, pre-polymerization of few monomers results in shrinkage, which decreases the system’s initial free volume and restricts the diffusion of monomers during the progression of the photopolymerization [65]. This phenomenon in VisCalor Bulk was investigated by Yang et al. who concluded, that pre-heating did not cause adverse effects through premature polymerization [28]. Other factors which may have been responsible for the observed temperature behavior of the FOB_55 could be further thermal side effects including evaporation of the reactants and thermal degradation of the photoinitiator [66]. In the case of VCB_65, 30 s pre-heating time was enough in the special dispenser, however, for FOB_55 pre-warming took more time in the EnaHeat Composite Heating Conditioner (15 min), which may influence the chemical condition of the components. Among the above-mentioned thermal side effects, a higher significance should be attributed to oxygen inhibition in the case of elevated RBC temperatures. As the temperature increases, the decrease in viscosity promotes oxygen penetration into the RBC. Oxygen reduces the extent of polymerization by scavenging on free radicals resulting in less reactive peroxy radicals and/or quenching of the excited triplet state of the initiator [43]. It is reasonable to assume a role also for a further FOB constituent, the so-called AFM, an addition-fragmentation chain transfer dimethacrylate monomer, which participates readily in network formation by copolymerizing with multifunctional methacrylates [67]. An AFM is a heteroatom (N or S or O) containing monomer with various vinyl activating groups which have been employed as chain transfer agents to reduce shrinkage stress [65,68]. However, chain-transfer reactions may also exert a retarding effect on the polymerization by increasing termination, especially at higher temperatures [65]. 

Although pre-heating did not increase monomer conversion in many cases, several studies have shown that the mechanical properties and marginal integrity of RBCs (including FOB and VCB as well) are satisfactory or better than those applied at room temperature [2,14,69,70]. In contrast, however, there are experiments concluding higher linear shrinkage of pre-heated RBCs and deterioration in marginal integrity [63,71], although, results of the available investigations show that the pre-heating has no significant impact on bond strength of RBC to dentin [72,73].

As our results confirmed, RBCs do not have a complete monomer to polymer conversion because of the condition-dependent kinetics of gelation, vitrification, immobilization, and steric isolation [74]. Incomplete conversion may result in the presence of unreacted monomer content within the polymer network which is partially or completely released short- or long-term [34,43]. Released monomers may depress the biocompatibility of the RBC by stimulating bacterial growth around the restoration leading to secondary caries development and may promote allergic reactions. Additionally, cytotoxic effects of monomers have been demonstrated [32]. Solubility and water sorption can accelerate the degradation and do harm to the mechanical/physical properties such as tensile-, flexural strength and wear [33]. To determine the quality and quantity of the residual monomers eluted from the investigated polymerized materials HPLC, as a generally applied investigative method, was used in our study [24,75,76]. Unreacted monomers can reduce the mechanical properties of the RBCs and their detection represents an important step for evaluating RBC biocompatibility [77]. 

During our experiment, aromatic (BisGMA) and aliphatic (TEGDMA, UDMA, and DDMA) dimethacrylate standard monomers were used to identify eluted monomers from the investigated RBCs. 

In the present study, 75% ethanol/water solvent was used to extract most of the examined unreacted monomers from the polymerized RBC specimens to identify monomer quantity. Besides the type of the solvent, the chemical nature of the matrix monomers and their combination, the degree of conversion, and the final network characteristics also play important roles in the quantity and quality of monomer elution from a certain RBC [24,34].

According to the manufacturer’s description, VCB is a BisGMA/aliphatic dimethacrylate based RBC but does not define in detail the aliphatic dimethacrylates. However, following HPLC measurements, TEGDMA, DDMA, and UDMA were detected as eluted aliphatic monomers from VCB. FOB is mainly an UDMA-based material, composed of both aliphatic and—as a BisGMA substitute—aromatic UDMA. However, released BisGMA was also detected during the HPLC measurements. Copolymers consisting of BisGMA and/or UDMA are crosslinked both chemically (C-C covalent bond) and physically (i.e., hydrogen bond). The latter determines the matrix viscosity, since the more numerous and stronger the hydrogen bonds are, the higher the viscosity of the system [56]. To create a sculptable RBC, the usage of these monomers is advantageous. 

Regarding the monomer release, our results showed elution to be strongly dependent on the material. 

In this study, significantly (two-fold) more UDMA and DDMA were released from the room temperature FOB_RT, meanwhile, VCB_RT samples leached almost three-fold more BisGMA. Pre-heating significantly decreased the monomer elution from FOB_55. There was no difference however in monomer elution between VCB_RT and VCB_65. The measured unreacted monomer release is in line with our results regarding the degree of monomer conversion in VCB_RT and VCB_65 since pre-heating did not change the DC on the top or bottom of VCB. On the other hand, the observed relationship between DC and monomer elution from FOB is contradictory. While the DC of the bottom surface decreased after pre-heating, the detected elution of unreacted monomers from FOB_55 samples was also lower. Although several studies have shown that the extent of leached unreacted monomer is correlated to the DC [4,78,79], the conversion degree does not necessarily correlate with the amount of free residual monomer, since the detected double bonds may remain as pendant groups bonded to the polymer structure and are not free to be released, however, may reduce the clinical success of the RBCs [56,80]. Probably, the above issue is the explanation for the lack of the expected relationship between the DC and monomer elution in the case of the pre-heated FOB_55. 

While the number of monomer elution studies from bulk-fill RBCs is extensive, data regarding the effect of pre-heating on monomer release both from conventional and bulk-fill RBCs is limited in the literature, hence, the discussion of this issue and comparison to other results are also restricted. Elution from bulk-fills was found to be comparable to that of conventional RBCs despite their increased increment thickness [34,81]. The quality and quantity of released resins are strongly material dependent and the amount of most of the eluted monomers is increased with time [24,82]. The monomer detected to be eluted in the highest amount was BisGMA from both VCB_RT and VCB_65, with the latter showing a significantly lower quantity. As it was previously mentioned, the extremely high viscosity of BisGMA limits the DC, leaving behind more unreacted monomers, which may release into the oral cavity. Admixing low molecular weight monomers, such as TEGDMA and DDMA, to BisGMA, can lower its viscosity, and via their synergistic effect can increase the rate of polymerization [83]. The released quantity of the latter two was very small both from VCB_RT and VCB_65. FOB, on the other hand, is a UDMA-based RBC. At present, UDMA is the only commercial alternative to the bisphenol A-based dental methacrylates [56]. Although, UDMA viscosity is lower than BisGMA, still high enough to require the addition of a reactive diluent, such as DDMA. Due to UDMA’s lower molecular weight in comparison to BisGMA, it is expected to show higher DC and lower unreacted monomer elution [84]. However, aside from aliphatic UDMA, FOB contains aromatic UDMA as well. Aromatic moiety and substitution symmetry play an important role in the steric hindrance, methacrylate group separation, limited conformational freedom, and increase of molecular stiffness. However, closer proximity of reactive groups facilitates the reaction-diffusion, which may lead to moderate DC and the planar geometry of benzene rings allows for building tighter structures [56]. Based on our results, the aliphatic UDMA release was moderate from FOB_RT and significantly lower from FOB_55, as a result of pre-heating. 

However, in our study, neither eluted AUDMA nor AFM was detected in the absence of the standards, as their exact chemical structure is a trade secret.

To the best of our knowledge, only one published article deals with the monomer elution from three pre-heated RBCs and found no effect of pre-cure temperature (68 °C) on the amount of leached UDMA, TEGDMA, and BisGMA [85]. Few available pieces of research reported that pre-heating of both conventional and bulk-fill RBCs did not influence cell viability, however, polymerized samples were used to examine the cytotoxicity without determination of the eluted monomers [63,83,86]. de Castro et al. investigated the sorption and solubility of RBC at higher pre-cure temperatures (60 °C) and concluded that longer curing times and higher temperatures led to lower values of sorption and solubility, but these differences were only significant for specific combinations of temperatures and curing times. [87].

The main limitation of this study may be the in vitro nature of the investigation. Although during sample preparation a conscious effort was made to simulate an isolated tooth by adjusting the PTFE mold temperature, the thermal conductivity of a natural tooth, its position in the oral cavity, the cavity configuration, thus the contact surfaces with the RBC are just a few mentioned factors, which may influence the results in vivo. Furthermore, the analysis of the elution of selected unreacted monomers (BisGMA, UDMA, TEGDMA, DDMA) will not provide an absolute measure of the quality of released components, since, among others, various monomers, like AUDMA, AFM, degraded compounds, initiator molecules, and fillers may also leach and compromise the RBC biocompatibility. A further limitation may be the limited number of the investigated high-viscosity bulk-fill RBCs, especially considering the strongly material-dependent results. The results cannot be extrapolated to other room temperature and pre-heated RBCs, since the composition has a strong influence on both DC and monomer elution and can vary from RBC to RBC.

## 5. Conclusions

Within the limitations of this in vitro study, the following conclusions can be stated:

(1) Significantly higher DC values were achieved on the top of the room temperature and pre-heated investigated bulk-fill RBCs than on the bottom.
(2) Room temperature VisCalor Bulk has lower DC% values both on the top and bottom compared to Filtek One Bulk.
(3) Pre-heating did not influence the DC of VisCalor Bulk, however, significantly decreased the DC at the bottom of Filtek One Bulk.
(4) Pre-heating had no effect on the monomer elution from VisCalor Bulk, but significantly decreased the monomer release from Filtek One Bulk.
(5) *Material* factor had a significant effect on each investigated variable, while *Temperature* factor and its interaction with *Material* is surface- (top vs. bottom) and monomer-dependent.

Based on the results, the following clinical significance can be deduced: While pre-heating had no beneficial effect on the degree of conversion neither of the thermoviscous VisCalor Bulk nor the contemporary bulk-fill RBC (Filtek One Bulk) the increased pre-cure temperature may decrease the elution of unreacted monomers from the RBCs.

## Figures and Tables

**Table 1:** Materials, manufactures, composition, and investigated pre-cure temperature of bulk-fill resin-based composites.

Material	Manufacturer	Pre-Cure Temperature	Code	Resin System	Filler	Filler Loading
VisCalor Bulk	Voco, Cuxhaven, Germany	Room temperature	VCB_RT	Bis-GMA, aliphatic dimethacrylates	Inorganic nanohybrid filler (not defined by the manufacturer)	83 wt%
		Pre-heated to 65 °C	VCB_65			
Filtek One Bulk-fill Restorative	3M ESPE, St. Paul, MN, USA	Room temperature	FOB_RT	AFM, UDMA, AUDMA, DDMA	20 nm silica, 4–11 nm zirconia, cluster Zr-silica, 0.1 µm ytterbium-trifluoride	58.5 vol% 76.5 wt%
		Pre-heated to 55 °C	FOB_55			

Abbreviations: BisGMA: bisphenol-A diglycidil ether dimethacrylate; AFM: addition-fragmentation monomer; UDMA: urethane dimethacrylate; AUDMA: aromatic urethane dimethacrylate; DDMA: 1,12-dodecane dimethacrylate; vol%: volume%; wt%: weight%.

**Table 2:** Differences in mean DC% (S.D.) between the top and bottom surfaces of the investigated materials polymerized at room temperature and pre-heated condition.

	Room Temperature					Pre-Heated				
	Top	Bottom	t-Value *	*p*-Value *	95% CI	Top	Bottom	t-Value *	*p*-Value *	95% CI
					Lower Upper					Lower Upper
**FOB**	63.0 (2.0)	51.8 (1.4)	31.7 (4)	<0.005	26.3 31.3	64.0 (1.4)	45.0 (1.9)	−17.2 (4)	<0.005	15.1 20.8
**VCB**	54.2 (2.9)	46.2 (1.4)	4.6 (4)	0.01	1.2 5.1	55.0 (1.3)	45.2 (4.1)	−25.1 (4)	<0.005	33.6 41.9

Abbreviations: DC, degree of conversion; FOB, Filtek One Bulk; VCB, Viscalor Bulk; CI, Confidence Interval. * Paired Samples Test.

**Table 3:** The relative effect size of factors Material, Temperature, and their interactions on the degree of conversion of the top and bottom surfaces of the investigated resin-based composites analyzed by General Linear Model and Partial Eta-Squared (ƞ^2^) statistics.

Factor	Degree of Conversion (DC)			
	Top Surface		Bottom Surface	
	*p* Value	Partial ƞ^2^	*p* Value	Partial ƞ^2^
Material	<0.001	0.86	0.028	0.27
Temperature	0.23	0.09	0.004	0.42
Material × Temperature	0.81	0.004	0.022	0.29

**Table 4:** Differences in monomer elution from Filtek Bulk One and Viscalor Bulk analyzed by Independent Samples T-test.

	Monomer	Resin Composite	Mean (S.D.)	t-Value (df)	*p*-Value	95% CI	
						Lower	Upper
Room temperature	TEGDMA	Filtek One Bulk	0				
		Viscalor Bulk	0.1 (0.01)				
	UDMA	Filtek One Bulk	0.33 (0.34)	20.57 (8)	<0.001	0.29	0.36
		Viscalor Bulk	0.01 (0.01)				
	BisGMA	Filtek One Bulk	0.22 (0.01)	−19.43 (8)	<0.001	−2.33	−1.84
		Viscalor Bulk	2.3 (0.24)				
	DDMA	Filtek One Bulk	0.26 (0.02)	13.08 (8)	<0.001	0.13	0.18
		Viscalor Bulk	0.1 (0.02)				
Pre-heated	TEGDMA	Filtek One Bulk	0				
		Viscalor Bulk	0.08 (0.01)				
	UDMA	Filtek One Bulk	0.17 (0.11)	2.92 (8)	0.02	0.03	0.27
		Viscalor Bulk	0.02 (0.03)				
	BisGMA	Filtek One Bulk	0.08 (0.08)	−10.9 (8)	<0.001	−2.29	−1.49
		Viscalor Bulk	1.98 (0.38)				
	DDMA	Filtek One Bulk	0.15 (0.09)	1.19 (8)	0.27	−0.05	0.16
		Viscalor Bulk	0.09 (0.04)				

Abbreviations: S.D., standard deviation; df, degree of freedom; CI, Confidence Interval; TEGDMA, triethylene glycol dimethacrylate; UDMA, urethane dimethacrylate; BisGMA, Bisphenol A diglycidil ether dimethacrylate; DDMA, dodecyl methacrylate.

**Table 5:** Differences in monomer elution at room temperature and pre-heated conditions were analyzed by Independent Samples T-test.

	Monomer	Temperature	Mean (S.D.)	t-Value (df)	*p*-Value	95% CI	
						Lower	Upper
Filtek One Bulk	TEGDMA	Room temperature	0				
		Pre-heated	0				
	UDMA	Room temperature	0.33 (0.34)	3.04 (8)	0.016	0.04	0.28
		Pre-heated	0.17 (0.11)				
	BisGMA	Room temperature	0.22 (0.01)	3.73 (8)	0.006	0.05	0.22
		Pre-heated	0.08 (0.08)				
	DDMA	Room temperature	0.26 (0.02)	2.6 (8)	0.03	0.01	0.21
		Pre-heated	0.15 (0.09)				
Viscalor Bulk	TEGDMA	Room temperature	0.1 (0.01)	3.12 (8)	0.014	0.01	0.04
		Pre-heated	0.08 (0.01)				
	UDMA	Room temperature	0.01 (0.01)	−0.87 (8)	0.41	−0.05	0.02
		Pre-heated	0.02 (0.03)				
	BisGMA	Room temperature	2.3 (0.24)	1.64 (8)	0.14	−0.13	0.79
		Pre-heated	1.98 (0.38)				
	DDMA	Room temperature	0.1 (0.02)	0.36 (8)	0.73	−0.04	0.05
		Pre-heated	0.09 (0.04)				

Abbreviations: S.D., standard deviation; df, degree of freedom; CI, Confidence Interval; TEGDMA, triethylene glycol dimethacrylate; UDMA, urethane dimethacrylate; BisGMA, Bisphenol A diglycidil ether dimethacrylate; DDMA, dodecyl methacrylate.

**Table 6:** The relative effect size of the factors Material, Temperature, and their interactions on the monomer elution from the investigated resin-based composites analyzed by General Linear Model and Partial Eta-Squared (ƞ^2^) statistics.

Factor	Monomer Elution							
	BisGMA		UDMA		TEGDMA		DDMA	
	*p* Value	Partial ƞ^2^	*p* Value	Partial ƞ^2^	*p* Value	Partial ƞ^2^	*p* Value	Partial ƞ^2^
Material	<0.001	0.96	<0.001	0.75			<0.001	0.49
Temperature	0.036	0.23	0.046	0.21	0.014	0.55	0.036	0.23
Material × Temperature	0.35	0.054	0.006	0.38			0.04	0.24

Abbreviations: BisGMA: bisphenol-A diglycidil ether dimethacrylate; UDMA: urethane dimethacrylate; TEGDMA: trietylene glycol dimethacrylate; DDMA: 1,12-dodecane dimethacrylate.

## Data Availability

Not applicable.
